# Influence of Titanium Dioxide Nanoparticles on Human Health and the Environment

**DOI:** 10.3390/nano11092354

**Published:** 2021-09-10

**Authors:** Mohammad Mamunur Rashid, Petra Forte Tavčer, Brigita Tomšič

**Affiliations:** Department of Textiles, Graphic Arts and Design, Faculty of Natural Sciences and Engineering, University of Ljubljana, Aškerčeva 12, 1000 Ljubljana, Slovenia; mamun444@gmail.com

**Keywords:** TiO_2_, titanium dioxide, human health, environment, toxicity, oxidative stress, biocompatibility, risk management

## Abstract

Nanotechnology has enabled tremendous breakthroughs in the development of materials and, nowadays, is well established in various economic fields. Among the various nanomaterials, TiO_2_ nanoparticles (NPs) occupy a special position, as they are distinguished by their high availability, high photocatalytic activity, and favorable price, which make them useful in the production of paints, plastics, paper, cosmetics, food, furniture, etc. In textiles, TiO_2_ NPs are widely used in chemical finishing processes to impart various protective functional properties to the fibers for the production of high-tech textile products with high added value. Such applications contribute to the overall consumption of TiO_2_ NPs, which gives rise to reasonable considerations about the impact of TiO_2_ NPs on human health and the environment, and debates regarding whether the extent of the benefits gained from the use of TiO_2_ NPs justifies the potential risks. In this study, different TiO_2_ NPs exposure modes are discussed, and their toxicity mechanisms—evaluated in various in vitro and in vivo studies—are briefly described, considering the molecular interactions with human health and the environment. In addition, in the conclusion of this study, the toxicity and biocompatibility of TiO_2_ NPs are discussed, along with relevant risk management strategies.

## 1. Introduction

TiO_2_ is one of the most abundant and widely used metal oxide nanomaterial in the world [1,2]. As an n-type semiconductor, with a band gap energy of 3.2–3.35 eV, depending on its crystal phase, TiO_2_ acts as an effective photocatalyst during the photocatalytic process for surface functionalization [3,4,5,6]. It has three crystalline structures: anatase, rutile, and brookite. Anatase is the most common type and rutile is the most stable form, while brookite is the rarest [7]. There is also amorphous TiO_2_, which is a non-crystalline form. TiO_2_ exists in various nanostructures, such as nanoparticles, nanotubes, nanorods and nanowires, nanofilms, nanosheets, and nanocoatings, with remarkable photocatalytic activity, which attract scientists to develop potential technological applications in multidisciplinary fields for industrial production [8].

TiO_2_ is widely used in food, paints, plastics, printing inks, papers, and biomedical and cosmetic products. Due to its unique properties, TiO_2_ is also advantageously used in textiles. For example, when applied to textile fibers, it confers various functionalities such as UV protection, photocatalytic self-cleaning and antimicrobial activity, electrical conductivity, and antistatic properties, as well as increased thermal stability. Moreover, it can also be used for solar energy conversion in the production of electronic textiles or wearable electronics. However, when tailoring its desired functionality, TiO_2_ is usually deposited on the surface of textile fibers, but due to its fairly poor adhesion to the fibers, a certain amount of TiO_2_ washes away, peels off, or wears off during the product’s lifetime. Therefore, such continuous leaching of TiO_2_ into the environment results in a potential threat to human health and the ecosystem.

Extensive use of TiO_2_ NPs reasonably raises significant concerns due to their potential nanotoxicity, induced by oxidative stress, which is influenced by ROS (Reactive Oxygen Species) formation on the surface of TiO_2_ NPs in the presence of UV light [9,10,11,12]. After the penetration of TiO_2_ NPs into the human body, inflammation, cytotoxicity, genotoxicity, immunotoxicity, and neurotoxicity may occur. Such nanotoxicity can damage the liver, kidneys, lungs, and skin. Special attention must be paid to production workers, in particular, who are in daily contact with high concentrations of TiO_2_ NPs. For instance, it has been proven that TiO_2_ NPs smaller than 20 nm cause a higher inflammatory response in human cells [13]. Furthermore, TiO_2_ NPs also pose certain damage to end users and researchers and, at lower concentrations, TiO_2_ NPs act as immunomodulatory agents, inducing inflammatory responses through specific interactions with immune system cells [14,15].

Undoubtedly, TiO_2_ is released into most ecosystems, including in the agricultural field, where its potential effects on soil properties, soil microflora, and plants are yet to be investigated. However, TiO_2_′s potential nanotoxicity to the aquatic ecosystem is currently under study, and has thus far revealed that TiO_2_ NPs negatively affect the suppression of the immune system of fish and invertebrates. Despite this, more research is needed to address the bioaccumulation profile of TiO_2_ and its associated biomagnification in the food web. To date, controversial results have been found when studying the toxicity of TiO_2_ NPs, which may be due to different particle sizes, doses, culture media, or test methods used [7,16]. Recently, green nanotechnology has been adopted by researchers to ensure the biocompatible and environmentally friendly use of TiO_2_ NPs by overcoming their drawbacks.

Due to the widespread use of TiO_2_, exposure to NPs by ingestion, inhalation, or sorption has become virtually inevitable. In the last five years, reviews have been published on the effects of TiO_2_ NPs in living organisms, focusing either on the toxicology of TiO_2_ NPs [1,2,11,17,18,19,20,21,22,23], safety concerns in various applications [24,25,26,27,28] and related impact on human health [28,29,30,31,32,33], or their effects on water/soil/environmental quality [34,35,36,37,38]. Such review studies are necessary to conclusively determine the environmental and human effects of TiO_2_ NPs. The purpose of this paper is to review recent advances in the potential health and environmental effects of TiO_2_ NPs in order to contribute to the establishment of a scientific basis for the safe application of TiO_2_ NP and to promote the sustainable development of nanotechnology. Accordingly, the main exposure modes of TiO_2_ NPs, their potential toxicity mechanisms on human cells through various signaling pathways along with the negotiable toxicity, the health effects of exposure to TiO_2_ NPs, the biocompatibility, and the environmental effects of TiO_2_ NPs are discussed in detail.

## 2. Modes of Exposure

### 2.1. Inhalation

Inhalation is the major route of nanoparticle penetration into the body [33,39]. When TiO_2_ NPs are inhaled, they are transported to various lung tissues, capillaries, airways, and alveoli and translocated to the heart, liver, nervous system, etc. [13]. Depending on the duration and concentration of inhalation, TiO_2_ NPs undergo short- and/or long-term clearance from bodily compartments. By inhalation, finer particles, such as anatase TiO_2_ NPs, have a more toxic effect than the comparatively coarser rutile form [40]. For a short-term, single-cycle inhalation threshold, a value of 3.5 mg/m^3^ is assumed for spray applications. For repeated inhalation, a threshold of 17 mg/m^3^ is assumed for an 8-h workday. In reality, however, the actual threshold is likely to be even higher. Nevertheless, it was shown that an inhalation concentration of up to 35 mg/m^3^ does not lead to chronic pulmonary overload [40].

### 2.2. Oral Route

TiO_2_ NPs can enter the blood via the oral route as they are used in foods, personal care products, sunscreens, and toothpaste. TiO_2_ NPs remain in the major organs for a long time and are eventually excreted in the stool. Absorbed TiO_2_ NPs in the liver, spleen, kidney, and lung tissues are responsible for possible nephrotoxicity and liver damage [41]. Even after the prolonged release of NPs, they can affect living organisms in the environment via biological pathways [42].

### 2.3. Dermal Route

Human skin has unique barrier properties that act against the penetration of TiO_2_ NPs into the skin. Several studies have found that TiO_2_ NPs cannot penetrate through the human dermis, even when their particle size is smaller than 100 nm [43]. Moreover, other studies have revealed that the TiO_2_ NPs that do manage to penetrate the skin do not exhibit toxicity under certain conditions [44].

### 2.4. Injection

TiO_2_ NPs can be developed as a highly efficient photothermal, medicinal, and synergistic agent to repair/remove diseased tissues such as cancer both in vitro and in vivo with negligible toxic properties, tissue damage, and kidney/liver dysfunction [45,46]. After injection of TiO_2_ NPs, endothelial cells can be disrupted, and they can proliferate and migrate despite the induction of toxicity and immunogenicity. It has been shown that TiO_2_ NPs injected into the blood during photothermal cancer therapy enrich the cancer tissue by diffusing, engulfing the cancer cells, and eventually killing them completely [47,48,49].

## 3. Toxicity of TiO_2_ NPs

There is a lack of information on the potential toxicity of TiO_2_ NPs. Indeed, despite numerous studies in this field, it is difficult to find separate studies using the same TiO_2_ NPs with the same experimental protocol to compare the results obtained. Nevertheless, the existing data support the potential toxicity of TiO_2_ NPs in humans, model vertebrates and invertebrates, plants, algae, and microorganisms. The mechanism of toxicity of TiO_2_ NPs to organisms can be outlined as follows: (i) production of reactive oxygen species (ROS) and formation of electron-hole pairs in the presence of light; (ii) binding of TiO_2_ NPs to the cell membrane via electrostatic interactions, resulting in cell wall damage and peroxidation of lipids in the cell membrane; and (iii) binding of TiO_2_ NPs to intracellular organelles and biological macromolecules [50].

### 3.1. Cytotoxicity

**Figure 1:**
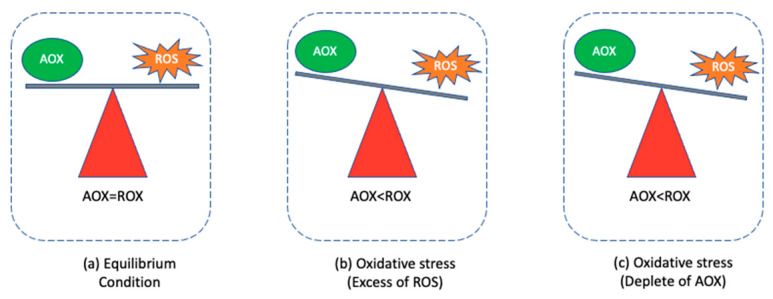
Equilibrium of ROS and antioxidants (AOX) (**a**) and their disequilibrium causing oxidative stress, either by an excess of ROS (**b**) or a deficiency of AOX (**c**).

It is well known that ROS (i.e., superoxide (O_2_^•−^), hydrogen peroxide (H_2_O_2_), and the hydroxyl radical (OH^−^)) are produced by aerobic organisms within the cell and are normally in equilibrium with antioxidant molecules (Figure 1a) [51]. The imbalance between ROS and antioxidants (AOX) caused by the excessive production of ROS or the depletion of antioxidant molecules leads to the occurrence of oxidative stress (Figure 1b,c) [51].

As for the negative biological effects, oxidative stress is the most important process involved in the formation of TiO_2_ NPs-induced ROS [52]. High TiO_2_ concentrations cause greater oxidative stress, which correlates with the increase in lipid peroxidation at the cell membrane after TiO_2_ NPs adsorption on the cell membrane. TiO_2_ NPs-induced ROS and lipid peroxidation damage the integrity of the cell wall and membrane, resulting in increased permeability [53], which allows TiO_2_ NPs to enter the cell. In addition to free diffusion, TiO_2_ NPs can also enter the cell through the process of endocytosis. While particles larger than 500 nm can be removed by phagocytes [50], smaller particles can be engulfed by a cell membrane vesicle and taken further into the cell. Accordingly, TiO_2_ NPs with a size of 25 nm and less have been taken up into human keratinocytes [54], as well as lung cells [52,55], lymphocytes [56], macrophages [57], keratinocytes [58], and hepatocytes [59] after endocytosis. It is also interesting to note the penetration ability of TiO_2_ particles with a size of 200 nm into red blood cells, which were chosen as a model for non-phagocytic cells. The results demonstrated the ability of the particles to penetrate the red blood cell membrane by a mechanism other than phagocytosis and endocytosis [60]. In this case, the penetration mechanism remained unexplained.

**Figure 2:**
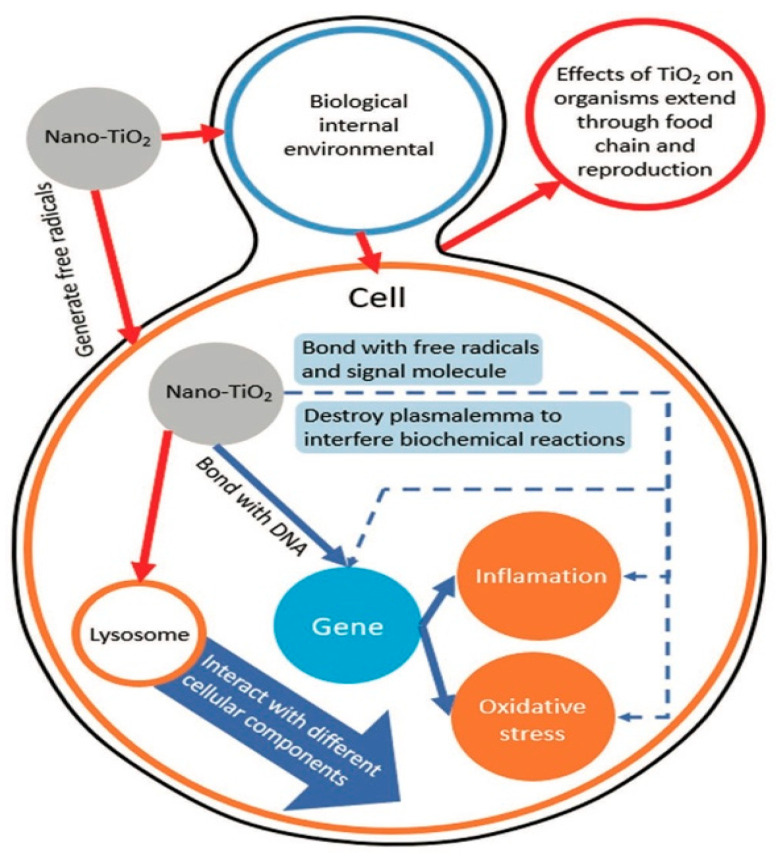
Toxicity mechanism of the reaction of TiO_2_ NPs with cells. Adapted with permission from [28]. Copyright, 2020 John Wiley & Sons, Inc.

Upon entrance of TiO_2_ NP into the cell, the internalized TiO_2_ NPs are transported to lysosomes, where they generate lysosomal stress and release cytosol that reacts with cellular components, resulting in DNA damage, DNA rearrangement, altered gene expression, oxidative stress, and inflammation (Figure 2) [28,56,61,62,63]. TiO_2_ NPs impair micromolecular functions by protein adsorption, blocking signaling pathways, and binding to DNA structure [64,65]. Undoubtedly, such damage affects cell viability [66] and is dose and time dependent [67,68].

**Figure 3:**
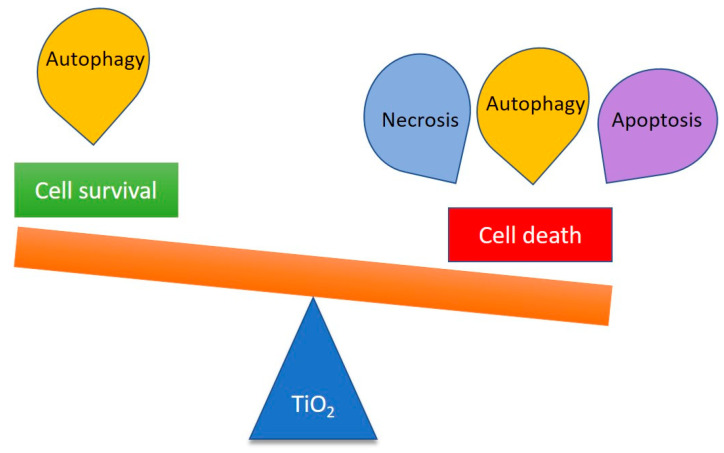
Influence of TiO_2_ NPs on the cell fate.

It should also be noted that the response of cells in the presence of TiO_2_ NPs is complex. Thus, the presence of TiO_2_ NPs can modulate different cell fates, including necrosis and apoptosis, which are regular cell death pathways, or autophagy, which leads to either cytoprotective mechanisms or cell death (Figure 3) [69]. TiO_2_ NP-induced autophagy can be exploited in new therapeutic pathway treatment of various diseases (see Section 6).

### 3.2. Genotoxicity

**Figure 4:**
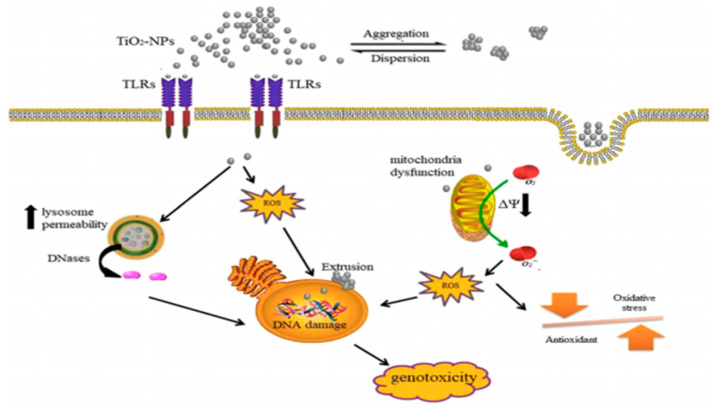
Mechanism of possible genotoxicity of TiO_2_ NPs in cells. Adapted with permission from [22]. Copyright, 2021 Sringer Nature.

The genotoxicity of TiO_2_ NPs has not yet been clarified as scientists do not have sufficient evidence of genotoxicity [70,71]. Genotoxicity refers to the ability of TiO_2_ NPs to disrupt genetic information by causing breaks, lesions, deletions, mis-segregation, or non-disjunction in the DNA, leading to gene mutations. In vitro testing methods, such as mammalian chromosomal aberration tests, cellular gene mutation tests and bacterial reverse mutation tests, are performed to measure genotoxicity [72,73]. In contrast, the in vivo comet assay and the in vivo micronuclei/chromosome aberration assay evaluate in vivo genotoxicity [74]. The genotoxic effect of TiO_2_ NPs on cells is mainly studied through the circulatory or respiratory system. Despite the crystallinity of TiO_2_ NPs, their genotoxicity mostly depends on their particle size. Smaller TiO_2_ NPs possess a stronger genotoxic effect than larger ones, as they easily penetrate into the nucleus and cytoplasm of the cell [74]. Larger agglomerations of TiO_2_ NPs cause DNA damage [75]. Several studies have shown the genotoxic and cytotoxic effects of TiO_2_ NPs on human amniotic epithelial cells [76], human lung fibroblasts [77], human lymphocytes [78], and human hepatoma HepG2 cells [79]. In vitro studies of cell-induced genotoxicity caused by TiO_2_ NPs due to DNA breakage and gene mutations are shown in Figure 4 [22].

Genotoxicity occurs via direct or indirect genotoxicity mechanisms. Namely, TiO_2_ NPs can enter the nucleus, inducing direct DNA damage through direct contact with DNA and chromosome, while indirect genotoxicity of TiO_2_ NPs results from the increased lysosomal release of DNases, the formation of nanoaggregates that can extrude nucleus or by ROS accumulation. Moreover, TiO_2_ NPs can also negatively influence the repair process of DNA. In vitro studies showed the genotoxicity of TiO_2_ NPs during short-term exposure, which may be triggered by smaller particle sizes and mixed phases of TiO_2_ NPs [22].

Research has provided contradictory results regarding TiO_2_ NPs’ genotoxicity [7,80]. Several studies found TiO_2_ NPs to have no genotoxic effect [7,57,80,81]. Brandão et al. examined TiO_2_ NP-induced genotoxicity in human lung, liver, glial, and neuron cells [82]. They found no genotoxicity, while TiO_2_ NPs were successfully internalized by the experimental cells.

## 4. Health Effects of Exposure to TiO_2_ NPs

### 4.1. Immune System

**Figure 5:**
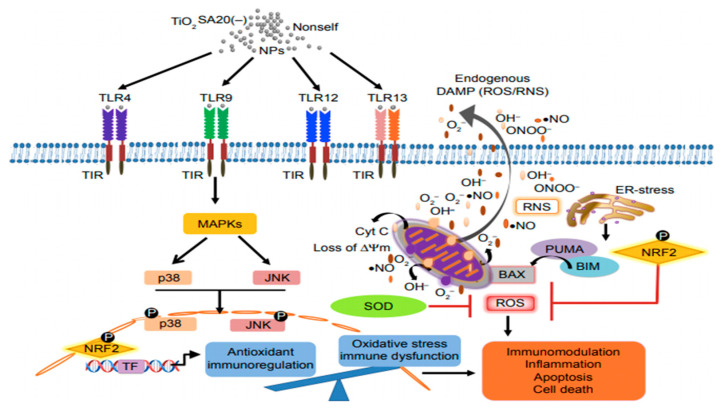
Mechanism of TiO_2_ NP-induced immunotoxicity in cells (TLR = Toll-like receptor; MAPK = mitogen-associated protein kinase; JNK = c-jun N-terminal kinase; NRF2 = nuclear factor erythroid 2 factor 2; and SOD = super oxide dismutase). Adapted with permission from [84]. Copyright, 2018 Dove Medical Press Limited.

The immune system defends the body against foreign antigens. If nanoparticles are recognized as foreign substances, they are eliminated by the immune system. If, on the other hand, the foreign substances are not recognized as a threat, they are either ignored or tolerated by the immune system [83]. The effects of TiO_2_ NPs on immune function are poorly documented. Underlying molecular mechanism by which TiO_2_ NPs influence the immune cell was studied in relation to TLRs, which are a subfamily of pattern recognition receptors, placed strategically on the cell surface and endosome of primary immune cell macrophages [84].TiO_2_-induced immunotoxicity was proven, which occurred through the activation of several receptors, which further activated specific signaling pathways to reduce the antioxidants through the formation of ROS. Upon increased ROS exposure, the mitochondrial membrane potential (Δψm) reduced, ultimately leading to apoptotic cell death, and inducing immunotoxicity through immune redox imbalance (Figure 5). However, in vivo experiments are essential to further validate TiO_2_ NPs influence on the immune system.

### 4.2. Neural System

**Figure 6:**
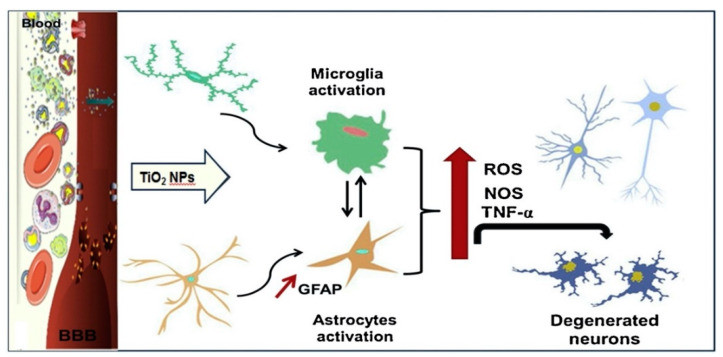
Mechanism of neurotoxicity of TiO_2_ NPs in cells. Adapted with permission from [89]. Copyright, 2020 Elsevier B.V.

TiO_2_ NPs can enter the brain regions by translocation across the blood–brain barrier or thenose–brain barrier, and progeny across the placental barrier, which may be the reason for the dysfunction and potential risks to the central nervous system [32,85,86,87]. Long-term exposure to TiO_2_ NPs can cause damages to the neurons and glial cells (U373), which may subsequently lead to neurotoxicity [32,88], also at low doses. In Figure 6, it is shown that the oral ingestion of TiO_2_ NPs can affect brain activities via increased oxidative stress, decreased antioxidant enzyme activity, and increased nitric oxide (NO) and ROS release (Figure 6) [89]. TiO_2_ NPs thus induced a neurotoxic damage accompanied by the increase in degenerated and apoptotic neurons in cerebral cortex.

### 4.3. Cardiovascular System

**Figure 7:**
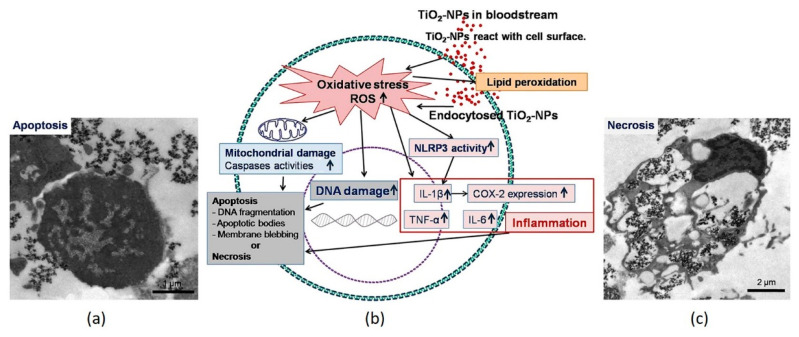
Cytotoxic mechanisms of peripheral blood mononuclear cells (PBMCs) investigated with TiO_2_ NPs (**b**). TEM images of apoptosis (**a**) and necrosis (**c**) of PBMCs. (COX-2 = cyclooxygenase-2; IL = interleukin; and TNF = tumor necrosis factor). Adapted with permission from [10]. Copyright, 2016 John Wiley & Sons, Inc.

TiO_2_ NPs of 100 nm significantly reduce mitochondrial ‘dehydrogenase activity’ in human lymphocyte cells [81]. Mitochondria-mediated apoptosis-based cell death due to DNA damage is induced by TiO_2_ NPs. When TiO_2_ NPs react with hemoglobin, they can weaken red blood cell oxygen transport. Kongseng et al. investigated the cytotoxic effects of TiO_2_ NPs on human blood cells, namely peripheral blood mononuclear cells (PBMCs). TiO_2_ NPs incubated for 24 h significantly suppressed cell viability and increased the formation of toxic mediators (Figure 7) [10]. At high TiO_2_ NP concentrations (≥ 25 μg mL^−1^), cell apoptosis and the ‘proinflammatory cytokine secretion’ of PBMCs increased due to oxidative stress caused by ROS. The effect of TiO_2_ NPs and bulk material in PBMCs, neopterin formation and tryptophan degradation was studied by Becker et al. [90]. Neopterin production was increased in both unstimulated and stimulated PBMCs, while tryptophan breakdown was suppressed, thus suggesting that the total effect of TiO_2_ NPs was strongly pro-inflammatory. Namely, in human body fluids, such response is detected in diseases such as infections and cancer, and is also parallel to the course of atherogenesis and neurodegeneration [90].

In contrast, no significant DNA damage caused to human peripheral blood lymphocytes treated with TiO_2_ NPs was observed by an alkaline assay. Only extremely higher concentrations (100 µg) can show the genotoxicity of TiO_2_ [91].

Very little research has been completed on the effect of TiO_2_ NPs on the cardiovascular system. Researchers suspect that deposited TiO_2_ NPs in the heart may lead to inflammatory responses, system malfunction, and cardiac damage. Depending on the type and duration of exposure, ultrafine TiO_2_ NPs could lead to an increase in heart rate, blood pressure, and cardiac muscle damage [32].

### 4.4. Respiratory System

The lungs are the main target system of environmental air pollution via TiO_2_ NPs [92]. TiO_2_-induced human lung epithelial cell injury (A549) and alveolar lung inflammation have been reported after inhalation in several studies [93,94,95]. These phenomena lead to lung dysfunction, as well as irreversible changes to the cells, resulting in fibrosis and tumor development. The lung toxicity and inflammatory effects are related to the properties of TiO_2_ NPs, such as their size, shape, crystallinity, agglomeration, and surface coating mode [96]. At this point it should be noted that the cytotoxicity of TiO_2_ NPs increased in the following order: amorphous > anatase > anatase/rutile; thus, amorphous TiO_2_ NPs possessed greater toxic effect than anatase/rutile TiO_2_ NPs [95]. Fresegna et al. studied the cellular responses of human alveolar A549 and bronchial BEAS-2B cells to measure the cytotoxic and inflammatory effects of TiO_2_ NPs in anatase and rutile forms [7]. They found that anatase TiO_2_ NPs exerted greater cytotoxicity on bronchial cells compared to rutile TiO_2_ NPs. On the other hand, a higher level of genotoxicity was observed on alveolar and bronchial cells treated with rutile TiO_2_ compared to anatase TiO_2_. When TiO_2_ NPs are pre-irradiated, they have a greater cytotoxic effect on human lung cells compared to non-irradiated TiO_2_ NPs [93].

### 4.5. Digestive System

Oral ingestion of TiO_2_ NPs causes them to enter the digestive system. The blood passes through the stomach and also passes through the liver. Significant concentrations of TiO_2_ NPs can lead to liver dysfunction, liver cell damage and even liver failure, hepatocyte dysfunction, superficial staining of cytoplasm, and osteoporosis after repeated exposure [40,92]. However, these results, carried out in mice or rats, are controversial, as no toxicity was observed in other studies.

### 4.6. Urinary System

The kidneys are one of the major organs that filter the blood, remove metabolic wastes, control the body’s extracellular fluid balance and electrolyte composition, and return the purified blood to the body. Chen et al. demonstrated an association between TiO_2_ NPs and kidney toxicity, suggesting that the kidney may be a major target or organ of exposure to nano-TiO_2_ via various routes into the body [92]. When cultured embryonic kidney cells were incubated with TiO_2_ NPs, no significant induction of DNA damage was observed. In this case, only the highest concentration of TiO_2_ NPs, equivalent to 100 μg/mL, elicited a significant genotoxic response, but it was concluded that such a high concentration of TiO_2_ is not environmentally relevant [91]. However, further studies on renal toxicity in humans due to exposure to TiO_2_ NPs need to be conducted.

### 4.7. Reproductive System

Based on the research conducted to date, the toxicity of TiO_2_ NPs to the reproductive system such as testes, ovaries, placenta, and fetal tissues in humans is unknown. TiO_2_ NPs cause adverse effects on hatching and affect reproduction in zebrafish, as well as pregnancy difficulties such as nanoparticle spread to fetal brain, fetal liver, and placenta in mice [13]. However, the research data are not sufficient to conclude the development of toxicity in the reproductive system of mammals, especially humans.

### 4.8. Dermal System

The penetration of TiO_2_ NPs through human skin has not been found in most studies [97,98,99]. TiO_2_ NPs do not cause ROS formation, cellular glutathione content, nor apoptosis when applied to human epidermal (A431) and keratinocytic (HaCaT) skin cells [100,101]. TiO_2_ NPs cause dermal toxicity only when they pass through healthy or damaged skin after long-term exposure [40]. However, the opposite phenomenon has been observed in several studies. Wright et al. showed that TiO_2_ NPs induce superoxide formation, caspase, and cell apoptosis in human keratinocyte cells (HaCaTs) in a dose-dependent manner. This causes cytotoxicity in HaCaT cells at 10^−4^–10^−5^ mol/L [100]. In fact, human skin is not only exposed to TiO_2_ NPs, but also external chemicals or stressors, such as UV light [102], which also damage human dermal fibroblasts. The Scientific Committee on Consumer Safety (SCCS) therefore suggested that TiO_2_ NPs should not be used in sunscreen formulations with high photocatalytic activity [1].

## 5. TiO_2_ NPs in the Environment (Ecotoxicity)

Ecotoxicity occurs when biological, physical, and chemical stressors affect living organisms in the ecosystem through altered biochemistry, physiology, and cellular interactions. The ecotoxic adverse effects of TiO_2_ NPs have been observed in water, aquatic animals, zebrafish gills, food, and aquatic environments [67]. TiO_2_ NPs influence the bacterial colonies in soil, reducing microbial biomass and diversity, thereby having a negative effect by changing the bacterial composition of the ecosystem [103,104]. In addition, terrestrial plants collect TiO_2_ NPs from the soil and store them in stems, leaves, and fruits, which promotes germination and root expansion [105,106].

### 5.1. TiO_2_ NPs in the Plant and Soil Environment

**Figure 8:**
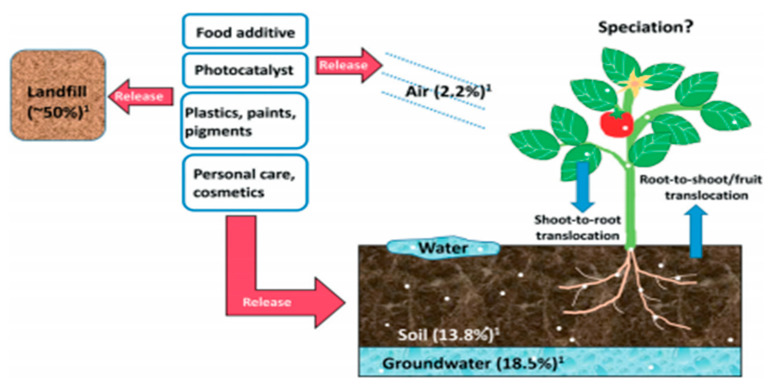
Uses and dispersion of TiO_2_ NPs into the environment and interaction with plants. Adapted with permission from [107]. Copyright, 2018 The Royal Society of Chemistry.

After the use of TiO_2_ NPs in various products, this inorganic nanomaterial is mostly abandoned, and a large quantity of TiO_2_ NPs is distributed to the environment, reaching the air, soil, water, and living organisms [54]. Figure 8 represents the use, release pathways, distribution, and interaction of TiO_2_ NPs to plants and the surrounding environment. TiO_2_ NPs used in pigments, food additives, and personal care products are released into the soil (13.8%), water (18.5%), and air (2.2%). Therefore, plants come into direct contact with TiO_2_ NPs through the soil, water, and air.

After the entry of TiO_2_ NPs via several pathways, soil properties—such as soil enzymes, microbial communities, nutritional elements, and pH—affect their behavior, mobility, and bioavailability, thereby determining the fate of TiO_2_ NPs. The interconnected soil properties/factors, such as soil type, pH, ionic strength, and organic matter, affect the transport of TiO_2_ NPs by changing their zeta potential, aggregation, surface charge, and van der Waals force [108,109]. The existence of microbes in the soil is vitally important for the decomposition and recycling of organic material. As a result, TiO_2_ NPs may alter the microbial population, diversity, and activity by changing the soil properties [103,110]. Low concentrations of TiO_2_ NPs also increase urea activity [111]. On the other hand, at extremely higher concentrations (1000 mg/L), TiO_2_ NPs decrease urea activity [112,113], affect the level of bacterial nitrogen, and reduce catalase, phosphatase, invertase, and peroxidase activities [103]. Reports suggests that TiO_2_ NPs also disrupt the gene expression of bacteria, resulting in decreasing nitrogen fixation and methane oxidation, which are essential for the decomposition of proteins and organic pollutants [81].

The interaction between plants and TiO_2_ NPs depends on their particle size, crystal phase, and surface coating [107]. Smaller TiO_2_ NPs (less than 30 nm) can enter into plant cells by reducing the size of the pores and the flow of water in corn [114] and wheat [115]. Investigations show that TiO_2_ NPs of 12, 22, and 25 nm can be translocated from the roots to the leaves [115]. The antioxidant stress is interrupted by TiO_2_ NPs in duckweed [116] and tomato [117]. TiO_2_ NPs also interrupt different parameters in raceme elm [118], onion [119], soybean [120], rice, spinach [121], and parsley [122]. As TiO_2_ NPs show photocatalytic activity under light irradiation, they can alter photosynthesis, metabolism, and gene expression within plants. Some investigations reported negative effects caused by TiO_2_ NPs on plant growth, whereas it was concluded that TiO_2_ NPs may cause some sensitive plant growth-promoting bacteria to disappear from soil. Accordingly, such impairment of the soil bacterial community composition may further affect ecosystem functioning [123,124].

### 5.2. TiO_2_ NPs in the Aquatic Environment

**Figure 9:**
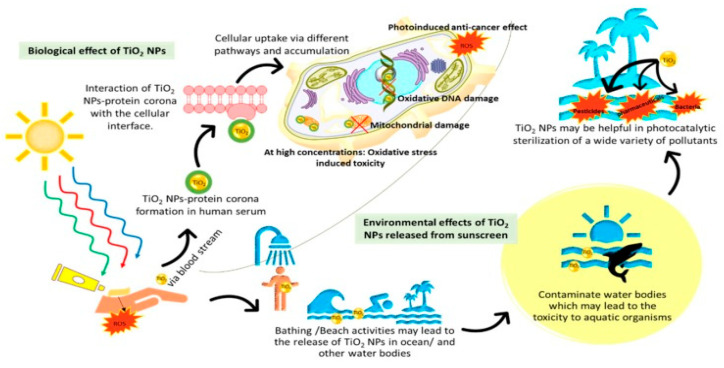
Environmental and biological effects of the TiO_2_ NPs. Adapted with permission from [24]. Copyright, 2019 MDPI AG.

The significantly increased leakage of TiO_2_ NPs into surface and marine water environments has a great impact on aquatic ecosystems. The related studies have been mainly concentrated in determining the behavior of TiO_2_ NP in marine environments, focusing mostly on marine plankton and benthos, with comparable contribution of papers, i.e., 42.1% and 44.7%, respectively, as well as in marine fish, with the smallest share of the research, i.e., 13.2% [125]. As TiO_2_ NPs are very reactive, they follow different transformation processes when they are released into the aquatic ecosystem. These transformation processes involve physical (agglomeration, aggregation, and sedimentation) interactions with TiO_2_ (adsorption), chemical (photochemical) interactions, and biological (biomodification) interactions. The ability of TiO_2_ NPs to enter in the aquatic organisms is worrying, as it leads to bioaccumulation in their cellular tissue. Accordingly, NPs negatively affect environmental food webs in three different major ecosystems—freshwater, marine, and terrestrial [24,126]. Bearing in mind that mammalians are at the top of the ecological food chain, the ecotoxicity induced by TiO_2_ NPs in the environment is easily converted into cytotoxicity in humans (Figure 9) [24].

Phytoplankton is the most dominant factor in marine ecosystems and the food web [127]. The interaction of TiO_2_ NPs with marine phytoplankton has therefore been investigated [127,128]. In an aquatic environment, TiO_2_ NPs may be adsorbed or diffused by the phytoplankton surface. TiO_2_ NP-mediated ROS may be diffused by the cell wall when the TiO_2_–plankton complex generates ‘ligand-to-metal charge transfer reactions’ [126]. After the aggregation and settling of TiO_2_ in marine environments, there is still a small fraction of TiO_2_ NP nanoparticles in the water column that may be hazardous to the living organisms in that water column. Thus, at higher TiO_2_ concentrations (>/20 mg/L), TiO_2_ NPs can significantly reduce *P. tricornutum* growth, one of the most widely used model organisms used in marine ecotoxicology studies [127]. Miller et al. demonstrated that, at low UV levels, TiO_2_ NPs’ photocatalytic activity can induce toxicity in marine phytoplankton [128]. However, in the absence of UV light, no effects were observed.

The toxicity of TiO_2_ NPs in marine mussels has been investigated in several studies [38,129,130,131,132,133,134]. Due to the filtrating behavior and bioaccumulation tendency of bivalve mollusks, they accumulate various pollutants, such as microalgae, sediments, bacteria, and contaminants, within their tissues [38]. However, no acute TiO_2_ NP toxicity was found in marine abalone at TiO_2_ NP concentrations from 0.1 to 10.0 mg/L [135]. In spite of the absence of toxicity, minor oxidative stress was induced. In another study, the combined effect of TiO_2_ NPs and ocean acidification was assessed in mussels [136]. A low pH increases the toxicity of TiO_2_ NPs and the impairment of feeding and metabolism was observed in mussels at different pH and concentrations.

In a different study, the TiO_2_ NP–fish interaction was examined at different TiO_2_ NP concentrations and exposure conditions [137]. TiO_2_ NPs of 0, 1, 10, and 100 mg/L were applied to fish for 96 h, resulting in no mortality or sublethal effects. Very few studies associated with TiO_2_ NPs have been conducted on fish. As such, more studies need to be conducted in order to understand the potentially cytotoxic effects of TiO_2_ NPs on fish.

Moreover, in aquatic environments, TiO_2_ NPs can interact with heavy metals (Cu, Zn, Cd, As, etc.) and toxic organics, resulting in the formation of a harmful environment that can alter the bioavailability of aquatic organisms [38,138,139]. If TiO_2_ NPs are exposed to arsenic (As), this can increase arsenic accumulation in aquatic animals and the human food web [139]. However, TiO_2_ NP–Cd interactions do not result in toxicity in Mediterranean mussels [140]. The immunotoxicity, genotoxicity, and neurotoxicity of TiO_2_ NPs in marine living organisms were reported by several researchers, with varying results depending on the particle size, exposure duration, exposure type, and stress factors [38,134,141,142,143,144,145].

## 6. Biocompatibility of TiO_2_ NPs

**Figure 10:**
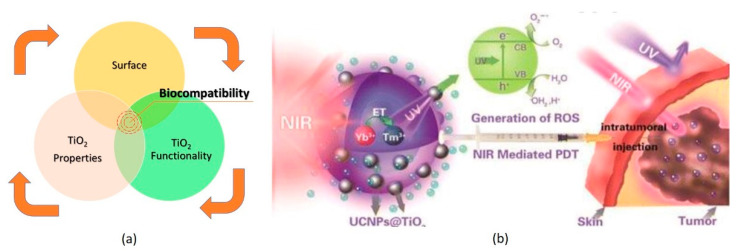
Biocompatibility of TiO_2_ NPs (**a**) and antitumor treatment by TiO_2_ NP ROS formation (**b**). Adapted with permission from [147]. Copyright, 2015 American Chemical Society.

The potential toxicity of the TiO_2_ NPs discussed above does not suggest that they are unsafe for humans or the environment. Depending on the size and shape of the nanoparticles, TiO_2_ NPs may be safe, due to their very low toxicity. When it comes to the issue of skin permeation, long-term skin exposure to TiO_2_ NPs can be harmful to humans if they overdose, which is almost impossible in everyday life [146]. Biocompatibility refers to the testing of TiO_2_ NPs for cytotoxicity, genotoxicity, immunotoxicity, systemic toxicity, hemocompatibility, pyrogenicity, and implantation, evaluated by ISO, ISO/TR 10993-22:2017 via in vitro and in vivo studies [47]. The evaluation of the toxicity and biocompatibility of TiO_2_ NPs is crucial to understanding the deleterious biological responses of the properties of TiO_2_ NPs, their functionalities, and their contact surfaces (Figure 10a).

In terms of the biocompatibility of TiO_2_ NPs, they are used in drug carrier biosensing, implants, and antibacterial activity [148]. Due to the spherical shape of TiO_2_, TiO_2_–cell interactions, TiO_2_′s biocompatibility, excellent drug release properties, and lower toxicity (than Al_2_O_3_ and SiO_2_) [148,149,150], TiO_2_ NPs are used in antitumor treatments (Figure 10b). Due to the cytotoxic ROS (O_2_^−^, OH^−^, H_2_O_2_)-based interaction between TiO_2_ NPs and specific cancer cell membranes, surface-functionalized biocompatible TiO_2_ NPs have recently been used for targeted cancer therapy [48]. TiO_2_ NPs have also been used in the biomedical field in photothermal therapy (PTT), photodynamic therapy (PDT), and sonodynamic therapy (SDT) for cancer treatment via the targeted, controlled, stimulus-driven delivery and release of cytotoxic anti-cancer agents [148]. Due to their low phototoxicity, biocompatibility and stable structure, TiO_2_ NPs have potential applications in phototherapy for the treatment of cancer cells [49,151]. When TiO_2_ NPs are exposed to light, an oxidative radical (ROS) is generated, which subsequently destroys the cell and cellular components, such as the lipids, proteins, carbohydrates, and nucleic acids in cancer cells [147,152].

Furthermore, several authors have reported that the toxicity of TiO_2_ NPs arises due to a ‘particle effect’ rather than a ‘chemical effect’ [40]. However, further studies are required to assess the nature, mechanisms, and effects of the toxicity of TiO_2_ NPs on humans and the surrounding environment.

## 7. Risk Management

### 7.1. Risk Following Oral Exposure

Considering the published studies, TiO_2_ NPs have no acute toxic effects after oral exposure. There are insufficient data on the repeated-dose toxicity of TiO_2_ NPs, so there is not believed to be any significant risk from oral exposure.

### 7.2. Risk Following Dermal Exposure

Based on the current data, short-term dermal exposure to TiO_2_ NPs has very little or no toxic effect on healthy skin. Further studies on long-term dermal exposure, as well as on damaged skin, are required in order to evaluate TiO_2_ NPs’ nanotoxicity on the skin [40].

### 7.3. Risk Following Inhalation

It is well established that smaller TiO_2_ NPs are more toxic than comparatively coarser TiO_2_ NPs. Anatase TiO_2_ is more toxic than rutile TiO_2_, with its crystalline structure. In terms of inhalation, particle size, surface area, crystalline structure, agglomeration, and exposure, time plays a crucial role in the toxicity of TiO_2_ NPs. Inhalation is a major problem in workplaces due to high concentrations of TiO_2_ NPs, as well as the fact that TiO_2_ NPs enter the body mainly through respiration.

**Figure 11:**
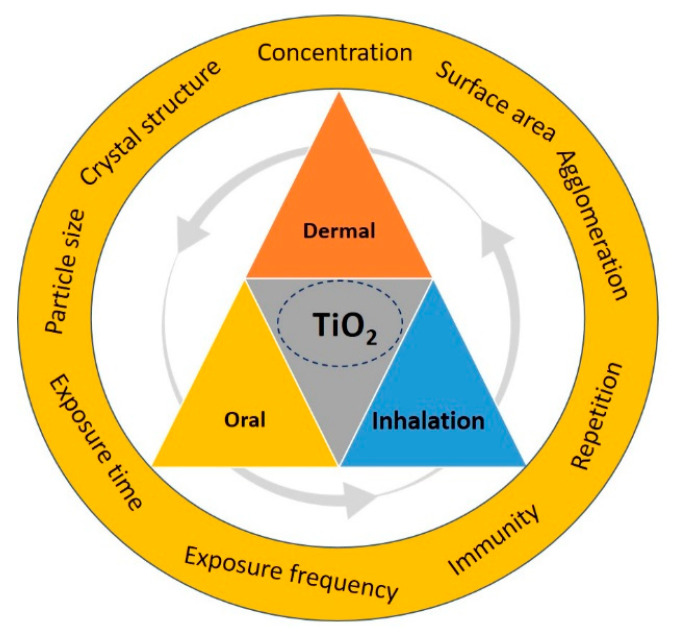
Risk management strategies for human exposure to TiO_2_ NPs via different exposure routes.

The establishment of risk management strategies is therefore crucial (Figure 11), particularly in relation to short-/long-term exposure, as well as the frequency and level of exposure; however, such strategies have not yet been developed. Regulations and legalization for controlling TiO_2_ NPs and other engineered NPs (ENPs) are unsure, living the interpretation of the scientific data challenging to the government, agencies, industry, and consumers. Recently, suggestions for the safe handling of TiO_2_ NPs and other engineered NPs (ENPs) were proposed by Besha et al. [153], highlighting the mitigation approaches to curtail the possible hazard effects of ENPs.

## 8. Conclusions and Future of TiO_2_ NPs in Textile Applications

TiO_2_ NPs are important photocatalysts that are implemented in various fields of application. The toxicity of TiO_2_ NPs to human health and the environment is still controversial. TiO_2_ NPs are classified as possibly carcinogenic to humans by the International Agency for Research on Cancer (IARC), and as a chemical risk to humans by the Workplace Hazardous Materials Information System (WHMIS) (in group D2A). At lower TiO_2_ NP concentrations, recent studies have found that TiO_2_ NPs are almost non-toxic, as no remarkable toxicity was observed [99,154,155]. However, TiO_2_/textile composites, for example, distinguished by remarkable functional properties [156], have no adverse effects on human skin, as superoxide and hydroxyl radicals are not able to damage human cells from the outside as they cannot enter the inner layer of the human skin [155,157]. As TiO_2_/rGO-coated cotton fabric is biocompatible, it does not cause cell membrane damage and cell proliferation, as textiles coated with TiO_2_ NPs do not exhibit cytotoxicity over 24 h of incubation [158]. With the exception of the other previously mentioned study, to the best of our current knowledge, no experiments have been conducted with TiO_2_ NP-modified textiles on human skin. Considering the toxicity and ecotoxic pathways described in in vivo and in vitro studies, TiO_2_ NPs are the safer option when compared to other metal oxide nanomaterials [148]. The nanotoxicity and contradictive biocompatibility of TiO_2_ NPs should be further investigated by researchers, whereas studies on long-term or accelerated effects of TiO_2_ need to be carried out in order to estimate precise implications of TiO_2_ NPs for human health and the surrounding environment. Therefore, careful consideration should be given to the benefits of TiO_2_, and the associated potential risks based on its intended use. Moreover, mitigating the toxicity of TiO_2_ NPs transferred from consumer products to the environment requires appropriate strategies and regulatory frameworks to protect humans and the environment. Accordingly, the establishment of international standard methods for the exact evaluation of risk–benefit assessments is a prerequisite to allowing the safe use of TiO_2_-functionalized materials.

## Data Availability

Data sharing not applicable.
